# Acute soft head syndrome in sickle cell disease: A rare and under-recognized complication with diagnostic challenges

**DOI:** 10.1016/j.radcr.2025.01.026

**Published:** 2025-01-28

**Authors:** Muhiddin Dervis, Adil Omer, Christopher Karakasis, David Mihal

**Affiliations:** a Cleveland Clinic, 9500 Euclid Ave, Cleveland, OH 44195, USA; b Ankara Yildirim Beyazit University, Ankara, Turkey

**Keywords:** Acute Soft Head Syndrome, Sickle Cell Disease, Subgaleal Hematoma, Epidural Hematoma

## Abstract

Acute Soft Head Syndrome (ASHS) is an exceptionally rare complication of sickle cell disease (SCD), characterized by scalp pain and swelling secondary to subgaleal hematoma formation. Distinguishing its nonspecific clinical features, which often mimic other SCD-related complications, poses a significant diagnostic challenge. Imaging plays a crucial role in differentiating ASHS from other potential diagnoses and in evaluating the presence of coexisting intracranial hemorrhage, a common complication associated with ASHS. This report presents a rare case of ASHS in a patient with SCD, highlighting the diagnostic complexities and underscoring the essential role of imaging in its evaluation and management.

## Introduction

Sickle cell disease (SCD) encompasses a group of autosomal recessive hemoglobinopathies characterized by the abnormal deformation of red blood cells (RBCs) into a sickle shape during periods of physiological stress. It is the 12th leading cause of mortality globally [1,2]. The clinical manifestations and symptom severity of SCD exhibit significant variability among affected individuals, influenced by genotype, environmental stressors such as infections, and access to medical care. Common complications of SCD include fatigue, anemia, and recurrent episodes of severe pain. In exceedingly rare cases, SCD may present with a serious, yet under-researched, complication known as Acute Soft Head Syndrome (ASHS). ASHS is characterized by the development of scalp pain and swelling, resulting from blood extravasation and hematoma formation beneath the galeal aponeurotic layer [3,4]. Diagnosing ASHS in SCD patients is particularly challenging, as headache and scalp swelling in this population can stem from various differential diagnoses [1,5].

## Case presentation

A 16-year-old male with a history of SCD presented to the emergency department with a chief complaint of a severe headache of maximal intensity, that had progressively worsened over the preceding 3 days. The patient's mother reported swelling on both sides of his head and described him as more fatigued and lethargic than usual, with frequent episodes of daytime somnolence. He reported experiencing photophobia and back pain but denied having fever, cough, dizziness, weakness, numbness, or tingling.

The patient's medical history was significant for multiple hospitalizations due to SCD-related complications, including recurrent pain crises, acute chest syndrome, and avascular necrosis of the left hip, for which he had undergone right hip arthroplasty. Family history was notable for maternal seizures.

On physical examination, the patient was afebrile with stable vital signs, and his neurological examination showed no focal deficits. He appeared to be in pain, reporting a 10/10 headache in the absence of recent trauma or infection. Bilateral scalp swelling was observed. Initial laboratory results revealed leukocytosis with a white blood cell count of 20.35 × 10⁹/L (reference range: 4.0–11.0 × 10⁹/L), a mild anemia with a hemoglobin level of 10.4 g/dL (reference range: 12–16 g/dL) and hematocrit of 29.3 % (reference range: 36–46 %). The reticulocyte count was elevated at 11 % (reference range: 0.5–1.5 %) with an absolute reticulocyte count of 0.369 × 10⁶/µL (reference range: 0.02–0.10 × 10⁶/µL), and the total bilirubin level was elevated at 3.7 mg/dL (reference range: 0.1–1.2 mg/dL), findings consistent with hemolysis.

Given his history of acute chest syndrome, a chest X-ray was performed, which was unremarkable with no new onset changes compared to an X-ray obtained one month earlier. To better evaluate the patient's severe headache and scalp swelling, a non-contrast CT scan was ordered to assess for potential intracranial pathology.

## CT without contrast

**Fig. 1:**
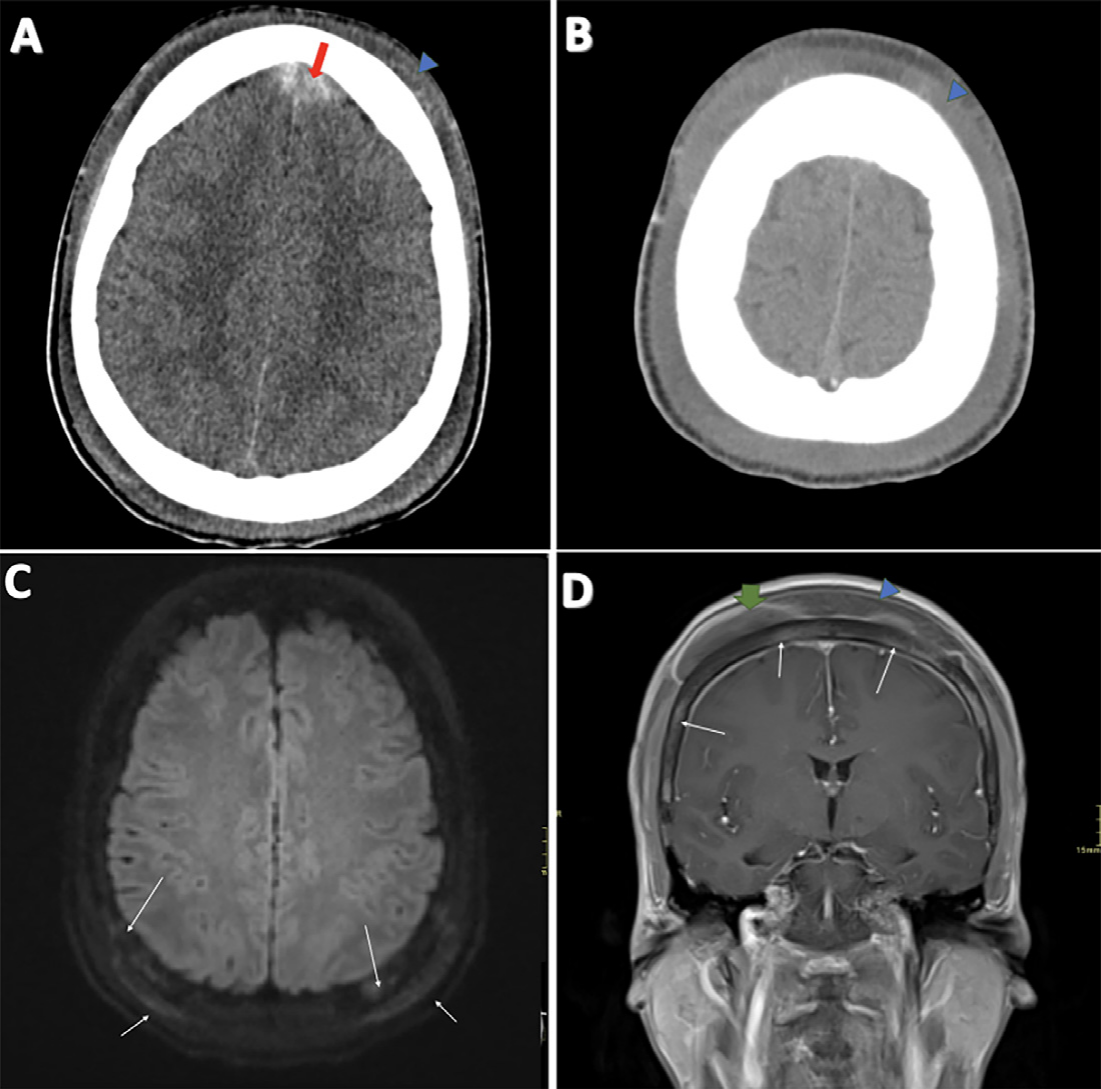
(A) Axial CT subdural window: The long, thick red arrow points to the extra-axial epidural collection. The blue arrowhead indicates subgaleal soft tissue edema. (B) Axial CT soft tissue window: The blue arrowhead indicates subgaleal soft tissue edema, appearing isodense on the axial CT soft tissue window. (C) Axial Diffusion-Weighted Imaging (DWI): The thin, long white arrows mark a region of occipital osteonecrosis. The thin, short white arrows point to the corresponding adjacent subgaleal hemorrhage. (D) Coronal T1WI Post-Contrast: The long, thin white arrows indicate calvarial heterogeneity with enhancement, suggesting areas of presumed bone infarction. The short, thick green arrows highlight pockets of subgaleal peripherally-enhancing collections with intrinsic T1 hyperintense signals. The blue arrowhead marks subgaleal soft tissue edema.

Serial axial brain images were obtained using non-contrast Computed Tomography (CT), extending from the vertex to the foramen magnum. The CT revealed a new onset of a 2.4 × 1.6 cm lesion with intermediate attenuation measuring 50 Hounsfield Units (HU), straddling and displacing the anterior cerebral falx (Fig. 1A). Minimal local mass effect was noted on the bilateral frontal lobes, with no significant adjacent edema or midline shift. No significant volume loss was observed in the brain parenchyma. Given the patient's history of SCD, the differential diagnosis included extramedullary hematopoiesis, dural venous sinus thrombosis, and meningioma. Furthermore, because of the lesion's attenuation, an extradural hematoma could not be ruled out. Additionally, extensive soft tissue enlargement was observed in the subgaleal scalp (Fig. 1B). These findings necessitated further evaluation with Magnetic Resonance Imaging (MRI) and Magnetic Resonance Venography (MRV) to better visualize the lesion and rule out associated pathologies.

### MRI brain WO/W contrast, MRV brain WO/W contrast

The MRI demonstrated heterogeneous marrow signal within the calvarium, characterized by T1/T2 hypointensity with mild diffusion restriction (Fig. 1C) and superimposed enhancement (Fig. 1D). Multifocal, well-circumscribed T1/T2 heterogeneous extraosseous lesions/collections were identified, demonstrating peripheral enhancement throughout the frontal and parietal scalp bilaterally (Fig. 1D). While the majority of these collections were extracranial, intracranial extraaxial collections were also observed along the right paramedian frontal epidural space. These findings were deemed unlikely to represent extramedullary hematopoiesis, which typically exhibits more pronounced and diffuse enhancement. They were also inconsistent with an abscess, which would show marked internal diffusion restriction. Instead, the collections displayed increased T1 hyperintensity and susceptibility on susceptibility-weighted imaging (Fig. 1A), compatible with hemorrhagic collections within the extracranial subgaleal space and intracranial epidural space, superimposed on areas of osteonecrosis and bone infarction.

**Fig. 2:**
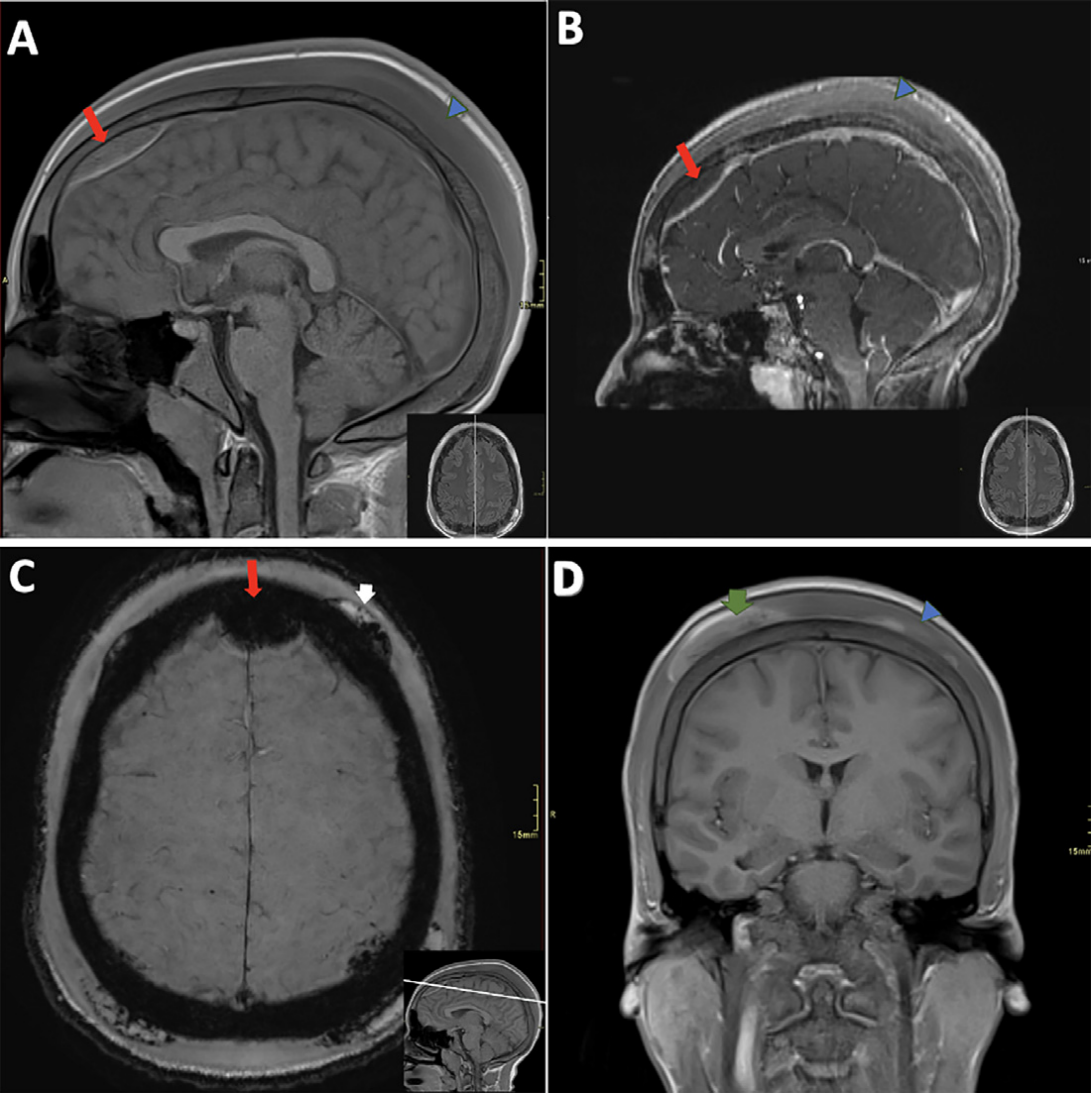
(A) Sagittal T1WI Pre-Contrast: The long, thick red arrow points to the epidural collection. The blue arrowhead highlights the thickened, edematous subgaleal tissue, with a T1 iso-intense signal. (B) Sagittal T1WI Post-Contrast: The long, thick red arrow indicates the epidural collection, with a non-enhancing T1 hyperintense signal. The blue arrowhead highlights the thickened and edematous subgaleal tissue, characterized by a T1 iso-intense signal with patchy, heterogeneous enhancement. (C) Axial Susceptibility-Weighted Imaging (SWI): The long, thick red arrow shows the epidural collection with an SWI hypointense signal. The short, thick white arrow highlights heterogeneous subgaleal collections with multiple confluent foci of susceptibility, surrounded by a T2 hyperintense signal, consistent with hemorrhage. D) Coronal T1WI Pre-Contrast: The short, thick green arrow highlights pockets of subgaleal collections with an intrinsic T1 hyperintense signal. The blue arrowhead indicates subgaleal soft tissue edema.

**Fig. 3:**
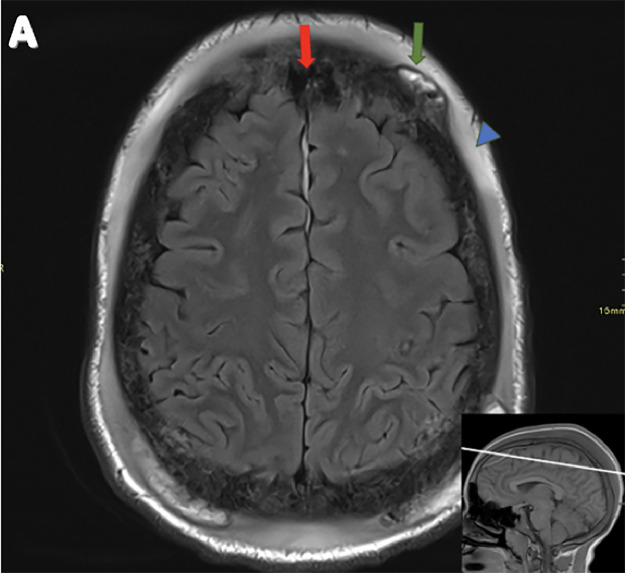
(A) Axial T2WI: The thick red arrow indicates an epidural collection with a T2WI hypointense signal, consistent with blood products. The thin green arrow highlights heterogeneous subgaleal collections with multiple confluent foci of susceptibility, surrounded by a T2WI hyperintense signal, consistent with blood products. The blue arrowhead marks thickened subgaleal and subcutaneous tissue with a T2WI hyperintense signal.

Mild to moderate stenosis was noted along the superior sagittal sinus, correlating with the regions of intracranial epidural collections, without evidence of dural sinus thrombosis. Extensive subgaleal fluid was observed in the scalp, appearing isointense on T1, hyperintense on T2, with mild, ill-defined enhancement, consistent with edema (Fig. 1, Fig. 2, Fig. 3). This constellation of findings is consistent with ASHS, an exceedingly rare complication in individuals with SCD.

The patient was admitted, and neurosurgical consultation determined that surgical intervention was not required. Treatment was initiated with intravenous fluids and analgesics, informed by prior case reports demonstrating significant symptom improvement with this approach. Throughout his hospitalization, head circumference was closely monitored to evaluate the stability of his condition. Intravenous antibiotics were administered to address the possibility of osteomyelitis but were discontinued after cultures returned negative. The patient's hospital course was complicated by acute chest syndrome, requiring transfer to the Pediatric Intensive Care Unit (PICU) for intubation, RBC exchange transfusion, and high-level supportive care. Additionally, he developed transient acute kidney injury, likely secondary to vancomycin and Non-Steroidal Anti-Inflammatory Drugs (NSAIDs) use, which resolved with conservative management. By day 10 of hospitalization, his condition had stabilized with significant pain improvement, enabling the discontinuation of sedation and successful extubation. Following a 13-day hospital stay, the patient was discharged with marked clinical improvement, including complete resolution of pain and stable respiratory function.

## Discussion

SCD refers to a group of inherited hemoglobin disorders characterized by the tendency of RBCs to polymerize and deform into a sickle shape under conditions of reduced oxygen availability [1]. Over recent years, mortality from SCD has risen significantly, placing it the 12th leading cause of death globally [2]. The hallmark symptom of SCD is recurrent episodes of severe pain, known as vaso-occlusive crises (VOCs). VOCs occur when sickled RBCs obstruct blood flow in small vessels. The lack of oxygen triggers intense pain, which can vary in severity, location, and duration. Other common complications include chronic fatigue, anemia, and an increased susceptibility to infections [1]. These profoundly affect the daily lives of those with SCD, influencing both their physical health and psychological well-being.

ASHS is a rare but serious complication of SCD, characterized by blood leakage and subsequent hematoma formation beneath the galeal aponeurotic layer, resulting in pronounced swelling of the head [[3], [4], [5]]. While the mechanisms underlying ASHS are not fully understood, several theories have been proposed. One theory posits that hypoxia in SCD triggers persistent extramedullary hematopoiesis in the skull, resulting in cortical bone thinning and fragility [3,10,11]. Another theory proposes that chronic hypoxia in these patients induces angiogenesis, leading to the development of fragile vascular networks. Combined with the hypoxia-induced elevation in cardiac output, this process may contribute to bone fractures and blood leakage into the subgaleal space [3,12]. Repeated VOCs are also thought to play a role, as they result in multiple small infarctions that progressively compromise the integrity of bone and periosteal structures. These infarctions can lead to vessel wall necrosis, which promotes blood extravasation into the subgaleal and epidural spaces, thereby contributing to the development of ASHS [3,9,13].

ASHS lacks pathognomonic radiologic features. MRI, widely recognized as the most sensitive diagnostic tool, typically demonstrates multiple non-enhancing calvarial lesions that appear hypointense on T1-Weighted Imaging (T1WI) and hyperintense on T2-Weighted Imaging (T2WI) [5,6]. These lesions are often accompanied by surrounding edema and may exhibit varying degrees of intracranial extension without significant mass effect [7]. Furthermore, MRI is essential for detecting associated intracranial abnormalities, including extra-axial collections and extramedullary hematopoiesis.

CT imaging, while less sensitive than MRI, remains valuable in diagnosing ASHS. CT typically demonstrates hyperdense scalp lesions extending across multiple regions of the skull, indicative of acute scalp and periorbital hematomas. Additionally, CT is crucial for excluding coexistent intracranial hemorrhage (ICH), which is a key consideration in managing patients with ASHS, as prior studies have frequently reported accompanying epidural hematomas [3,4,8,9].

ASHS is an extremely rare complication, with limited data available on its global prevalence and clinical characteristics. Diagnosis is particularly challenging due to this scarcity of literature and its overlap or co-occurrence with other SCD-related conditions, such as extramedullary hematopoiesis. However, accurate diagnosis is crucial, as ASHS is typically treated conservatively, and incorrect diagnosis may lead to unnecessary surgical interventions.

## Conclusion

ASHS is a rare and often underrecognized complication of SCD. This case highlights the need for increased awareness of ASHS among clinicians and radiologists to ensure timely and accurate diagnosis, avoid unnecessary surgical interventions, and improve patient outcomes. Further research and documentation are essential to enhance our understanding and management of this condition.

## Declaration of generative AI and AI-assisted technologies in the writing process

During the preparation of this work the author(s) used (Chat GPT) in order to enhance language and readability. After using this tool/service, the author(s) reviewed and edited the content as needed and take(s) full responsibility for the content of the publication.

## Patient consent

Written, informed consent for the publication of this case report was obtained from the patient's legal guardian. The patient was informed about the nature of the case report and agreed to the use of their medical information for educational and research purposes, with assurance that personal identifiers will remain confidential.
